# Adipose-derived stromal vascular fraction prevent bone bridge formation on growth plate injury in rat (in vivo studies) an experimental research

**DOI:** 10.1016/j.amsu.2020.09.026

**Published:** 2020-09-28

**Authors:** Panji Sananta, Rahaditya I Gede Made Oka, Prof Respati Suryanto Dradjat, Heri Suroto, Edi Mustamsir, Umi Kalsum, Sri Andarini

**Affiliations:** a Doctoral Program of Medical Science, Faculty of Medicine, Universitas Brawijaya and Orthopaedic and Traumatology Department, Faculty of Medicine, Universitas Brawijaya, Saiful Anwar Hospital, Malang, East Java, Jalan Jaksa Agung Suprapto No. 2, Klojen, Kota Malang, Jawa Timur, 65112, Indonesia; b Orthopaedic and Traumatology Department, Faculty of Medicine, Universitas Brawijaya Saiful Anwar Hospital, Jalan Jaksa Agung Suprapto No. 2, Klojen, Kota Malang, Jawa Timur, 65112, Indonesia; c Orthopaedic and Traumatology Department, Faculty of Medicine, Universitas Brawijaya, Pediatric Division, Saiful Anwar Hospital, Jalan Jaksa Agung Suprapto No. 2, Klojen, Kota Malang, Jawa Timur, 65112, Indonesia; d Orthopaedic and Traumatology Department, Faculty of Medicine, Universitas Airlangga, Hand and MicroSurgery Division, Sutomo General Hospital, Jl. Mayjen Prof. Dr. Moestopo, No.6-8, Airlangga,. Kec. Gubeng, Kota SBY, Jawa Timur, 60286, Indonesia; e Department of Orthopaedic and Traumatology, Lower Extremity and Adult Reconstruction Division, Saiful Anwar Hospital, Jalan Jaksa Agung Suprapto No. 2, Klojen, Kota Malang, Jawa Timur, 65112, Indonesia; f Department of Pharmacology, Faculty of Medicine, Universitas Brawijaya, Jalan Veteran no 2, Lowokwaru, Kota Malang, Jawa Timur, 65112, Indonesia; g Department of Public Health, Faculty of Medicine, Universitas Brawijaya, Jalan Veteran no 2, Lowokwaru, Kota Malang, Jawa Timur, 65112, Indonesia

**Keywords:** Adipose-derived stromal vascular fraction, Growth plate injury, Tissue engineering

## Abstract

The growth plate is cartilage tissue found at the end of long bones in children, responsible for longitudinal bone growth. Injuries to the growth plate cartilage often lead to unwanted bony repair, resulting in growth disturbances such as limb length discrepancy and angulation deformity in children. There is currently no clinical treatment that can fully repair an injured growth plate. Tissue engineering is promising for regeneration of growth plate. Adipose-derived stromal vascular fraction highlight the promising potential as tissue engineering therapy for inducing regeneration of injured growth plate and able to reduce the formation of bony repair that can lead to deformity and limb length discrepancy. Using an animal model of growth plate injury, bone bridge formation is evaluated after 28 days using Enzyme-linked Immunoassay, radiology, histopathology and Immunofloresence examination. Radiological analyses performed by evaluation of grey value using ImageJ software and diameter bone bridge measured from the end to end distance between uninjured growth plate evaluated by histopatology examination. Enzyme-linked Immunoassay and immunofloresence are used to evaluate chondrocyte and chondrogenic marker within the defect. The result shows in group with Adipose-derived stromal vascular fraction have a significant lower bone bridge formation compare to positive control group. This current study represents the first work that has utilized this animal model to investigate whether Adipose-derived stromal vascular fraction can be used to initiate regeneration at the injured growth plate.

## Highlights

• Injuries to the growth plate cartilage often lead to unwanted bony repair, resulting in growth disturbances
• There is currently no clinical treatment that can fully repair an injured growth plate
• Tissue engineering is promising for regeneration of growth plate.
• Adipose-derived stromal vascular fraction (AD-SVF) highlight the promising potential as tissue engineering therapy
• AD-SVF decrease bone bridge formation on growth plate injuries model on Rat

## 1 Introduction

The growth plate is a cartilaginous tissue located at the proximal and distal ends of the long bones of children, and is responsible for longitudinal bone growth and regulated process called endochondral ossification [1,2]. The growth plate is a common site for trauma injury and being the most fragile area of the growing long bones in children with up to 20% of children's bone fractures involving growth plate damage [3]. The Salter–Harris classification system has been used to determine and predict the outcome, prognostic factor and severity of a growth plate injury. This classification system divides growth plate injury into five types. Briefly, type 1 and 2 injuries (with type 2 being the most common injury type) do not interrupt the epiphyseal blood supply and thus will usually heal by itself with no overall growth problems. On the other hand, 30% of growth plate-related injuries are of types 3, 4 or 5, which affect all of the growth plate layer and also the blood supply. As avascular and alymphatic nature of cartilage, growth plate has limited ability to regenerate itself once damaged and has a poor regenerative capacity. Injury to growth plate cartilage often results in an undesirable repair response mechanism and may lead to bone bridge formation. Therefore, injury to the growth plate can represent a significant problem for the developing long bone particularly in a young child who has a significant remaining growth period, with up to 30% of growth plate injuries resulting in growth arrest and thus a limb length discrepancy and/or an angular deformity [2](4). Currently, the pathophysiology for the bony repair and bone growth defects remains unclear, there is no cell-based biological based therapy to prevent bone bridge formation and regenerate the damaged growth plate cartilage in clinical practice.

A greater understanding of these biological mechanisms could lead to more effective therapeutic interventions for children suffering from growth plate injuries. An earlier study identified four different phases of injury responses in a rat growth plate injury model (28) - the inflammatory, fibro genic, osteogenic and bone bridge maturation remodeling responses occurring during days 1–3, 3–7, 7–14, 10–28, respectively [2]. Following the initial inflammatory phase, a fibrogenic phase is apparent at the growth plate injury site. In this phase, influx of vimentin (mesenchymal cell marker)-immunopositive mesenchymal cells, a response which is similar to the infiltration of mesenchymal cells following the inflammatory phase at bone fracture sites. Due to the limited intrinsic regeneration capacity of cartilage tissues, there has been an increasingly expanding interest in using tissue engineering and stem cells for cartilage repair and growth plate regeneration. Since tissue engineering is a complex process involving the acquisition and cultivation of adequate cells, growth or differentiation-inducing stimuli and scaffolds supporting tissue formation, researchers worldwide have been working to prepare the 3 fundamental components for the successful tissue engineering for cartilage repair: (a) the right set of viable MSCs; (b) appropriate soluble inductive signal molecules or growth factors that are chondrogenic; and (c) appropriate biological or artificial carriers or delivery systems or matrix scaffolds that support cartilage formation in vivo [3].

Adipose-derived stem/stromal cells (ADSCs) were first characterized in 2001, and have since been widely studied and used as a major source of cells with regenerative potential, with characteristics similar to that of mesenchymal stem/stromal cells (MSCs). Within the AD-SVF is a collection of heterogeneous cells and components, most notably: MSC, HSC, Treg Cells, Pericyte-EC, mast-cells, complex microvascular beds (fibroblasts, WBC, dendritic cells, intra-adventitial smooth muscular-like cells, etc.), and extracellular matrix [5](6). In order to elicit a more potent chondrogenic differentiation response from MSCs, the combinational use of growth factors and scaffold can often increase chondrogenesis. Recent study for using SVF for tendon healing by Polly in 2019 shows that gene expression for IGF-I, TGF-β1, and FGF-2 was significantly higher in SVF cells than in cultured ASC. Those growth factor has main role in chondrogenesis. The effects of each growth factor to mesenchymal stem cell in chondrogenesis process are TGF-β1 Increases proliferation and ECM production also downregulates collagen type 1 gene expression; IGF-I Stimulates cell proliferation, increases expression of ECM, additive effect when combined with TGF-β1; Increases Proteoglycan synthesis and increases cell proliferation [7], [8].

Thus, it is important to understand whether the AD-SVF can be used as cell-based biological based therapy in preventing bone bridge formation in growth plate injury. The aim of this study is to evaluate the potential of Adipose-derived Stromal vascular fraction as a tissue engineering-based therapies for preventing bony bridge formation on growth plate injury model in rat. We used rat because Erickson et al., in 2017 has already established Growth Plate injury model in rat and repair mechanisms of the injury itself [9].

## 2 Material and methods

### 2.1 Study Design and animal model

**Fig. 1:**
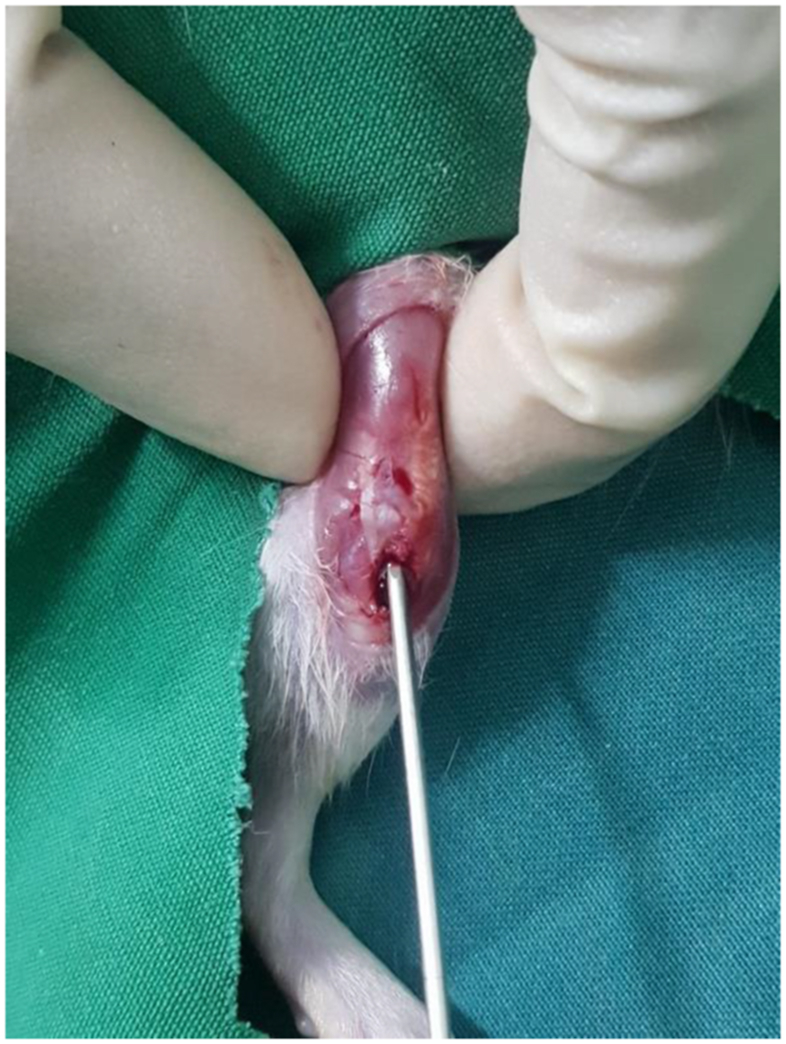
Overview of Surgical Procedure. Location of several anatomical markers used to create a successful growth plate injury. The knee capsule is immediately posterior to the kneecap (white), separating the tibia from the femur. The proximal growth plate is a mostly flat plane, except for the anterior quarter that forms a diagonal plane. Incision through the anterior-medial aspect of the tibial soft tissues to access the cortical bone. Location of the cortical window using alignment with the distal semitendinosus insertion as a reference point. Evaluating the depth of the injury by aligning the bevel on the kirschner wire with the cortical window.

Study Design for this research is experimental laboratories using post-test only control group and the work has been reported in line with the ARRIVE statement [10]. A total 18 male Rat (Rattus novergicus strain Wistar), approximately 6 weeks old, skeletal immature, with a mean weight ± 200 g and 5 adult female Rat as source for adipose tissue, were used in this study. Among them, 5 adult female rat were sacrificed to obtain adipose tissue from retroperitoneal fat as a source for Stromal Vascular Fraction. While the other 18 male rat were randomly allocated to the following three groups: Control negative group (without injured to growth plate, n = 6), Control positive group (untreated sample after growth plate injury, *n* = 6); Intervention group (treated with 0.8 ml AD-SVF *n* = 6); . Growth plate injury was made by using anatomical landmark; semitendinosus insertion, joint capsule and growth plate angle according to previously described methods (Fig. 1) [9]. For intervention group, SVF were added into the defect and closed the defect using bone wax. After surgery, all rat were permitted to exercise freely during daily life. At 28 days after intervention, all rat were euthanized to evaluate bone bridge formation on proximal tibia by performing imaging and histological analyses. All animal protocols were approved by the Ethics Committee of Faculty of Medicine Brawijaya University and all subsequent experiments were performed in accordance with relevant guidelines and regulations. This method has already established by Erickson in 2017 [9].

### 2.2 Isolation of AD-SVF

Adipose tissue (24.02 ± 9.31 g) was collected from 5 adult female rat to harvest allogous SVF as previously described. Briefly, adipose tissues were first washed three times in phosphate-buffered saline (PBS) to remove red blood cells and tissue debris; then, fat was digested with 0.2% collagenases I (Sigma C5138) (Sigma-Aldrich, St. Louis, MO, USA) at 37 °C for 1.5 h. SVF pellets were obtained by centrifugation at 425*g* for 5 min, followed by resuspension. The average yield of SVF was 2 × 10^6^ mononuclear cells per 1 g of rat adipose tissue.

### 2.3 Radiographic analysis using ImageJ software

To evaluate bone bridge formation using imaging analyses, after 28 days we sacrifice both groups and performing plain radiography for tibia. Choosing a method for radiological examination, the ALARA principle has to be considered to minimize radiation exposure dose. Regarding digitally processed radiographic, measurements of grey values is an easy feature of most graphic analysis software. The grey values are related to the absorption of x-rays, the radiologic density of a certain tissue. The grey values are saved in an 8-bit color space. Every pixel obtains a value between 0 and 255 in which 0 stands for black, low radiologic density and 255 for white, total x-ray absorption. Analyzing mean grey values, it has to be reconsidered, that an uncompressed file format like TIFF is being used, as compressed formats like JPG are summarizing areas of similar grey values, which leads to a loss of contrast, and significant changes in mean grey values. The measurement of the mean grey values was performed with the software ImageJ 1.44p (Wayne Rasband, National Institute of Health, USA). ImageJ is a free of charge software which has been used in medical and biological image analysis for a long time and has a wide range of analysis functions. Image files can be opened and Regions of Interests (ROI) can be created automatically or by freehand selection. The ROIs can be saved and transferred to other radiographic images and edited afterwards to different projection conditions.

### 2.4 Histological analysis

Proximal tibia samples were collected and fixed in 4% paraformaldehyde for one week, followed by decalcification in ethylenediaminetetraacetic acid (EDTA)-buffered saline solution. Samples were embedded in paraffin for sectioning (6 μm for each section). To assess the general morphology of the articular cartilage samples, sections obtained from rat in each group underwent histological staining, including H&E (hematoxylin & eosin) staining. Bone bridge diameter was quantitatively measured using tools on dotslide application in 8 times magnification. The defect of growth plate was qualitatively assessed by one blinded experts (Dr. EN) in 40 times magnification, to evaluate the cell characteristic that regenerate within the defect.

### 2.5 ELISA analysis

Growth plate of proximal tibia were collected by excise the cartilage of growth plate from the adjacent bone. Tissue samples should be collected, weighed, and added to 9× volume of lysis buffer (50 mM Tris-HCL with 2 mM EDTA, pH 7.4). Tissue may be homogenized using a Potter-Elvehjem homogenizer attached to a variable-speed drill or a tissuemizer. During the homogenization process, the tube should be submersed in an ice bath to maintain the sample at 4 °C. Following homogenization, the tissue preparation is centrifuged for 2 min in a microfuge at 13,000×*g*. After the tissue homogenates is ready, add sample and ELISA (SOX9) reagent into each well. Incubate for 1 h at 37 °C and add stop solution and colour will develop.

### 2.6 Immunofloresence analysis

Proximal tibial growth plate cartilages of rats after 28 days were excised, fixed and processed using decalcification as same as histological analysis described above without staining. Slides were incubated at 60 °C for 60 min then pre-treated using Xylol, Ethanol absolute, ethanol 90%, ethanol 80%, ethanol 70% and sterile water respectively. Slide retrieves the antigen with citrate buffer then stained for unlabelled osteocalcin and collagen type 2 (primary antibody) in 4 °C for 1 night. Incubation for secondary antibody in room temperature for 30 min, continued with DAPI staining 1:1000 and evaluate under fluorescence microscopes.

### 2.7 Statistical analysis

The data are expressed as the mean ± SE. Differences among groups were tested for statistical significance by performing Tukey followed by Pearson correlation. *P*-values less than 0.05 were considered statistically significant.

## 3 Results

**Fig. 2:**
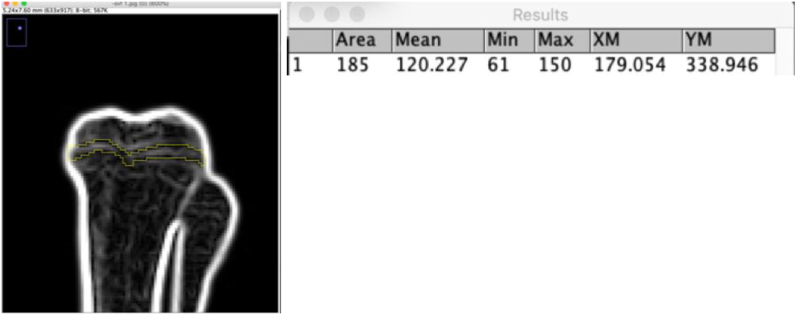
Evaluation of Mean Grey Values using ImageJ Software. Regio of Interest (ROI) was determined within the growth plate area. ROI representing the defect region. On the right hand the results of the measurements are shown.

All of the sample was in good condition and there is no behavioral changes. Analyzing mean grey values are summarizing areas of similar grey values, which leads to a loss of contrast, and significant changes in mean grey values. The measurement of the mean grey values was performed with the software ImageJ 1.44p (Fig. 2).

From descriptive analyses demonstrate mean grey value from control positive group revealed a highest mark (121.763 ± 4.831) compared to negative control group and intervention group (AD-SVF treatment) (76.141 ± 6.737) (91.231 ± 6.616) with the highest mean grey value was 127.007 (control positive group) and the lowest mean grey values was 63,590 (negative control group) as seen on Table 1 and Chart 1. The grey values are related to the absorption of x-rays, the radiologic density of a certain tissue which it means the untreated samples (positive control) have a higher radiological density within the growth plate compare to the negative control group and treated group. Tukey test revealed p values was 0,000, less than 0.05, which means there was a significant difference of mean grey values between positive control samples compares to negative control and treated SVF group. But, there was no significance difference between negative control group and treated SVF group.

**Table 1:** Descriptive analyses of mean grey values between three different group.

Parameter	Group	N	Mean	Std. Deviation	Minimum	Maximum
Mean grey value	Negative control	6	76.141	6.737	63.590	82.506
	Positive control	6	121.763	4.831	113.737	127.007
	SVF	6	91.231	6.616	81.552	98.967

Chart 1. Demonstrates untreated group (positive control) have higher mean grey values which means a higher density within the growth plate in comparation with negative control group and treated group (SVF).

Histological examination of the untreated samples revealed extensive bone formation within the defects (Fig. 3a). AD-SVF group to the defect site, on the other hand, resulted in less bone bridge repair (Fig. 3b). Diameter of bone bridge was measured from end to end between the uninjured growth plate. Descriptive analyzes revealed that mean of bone bridge diameter from positive control group was higher (1.256.83 μm ± 429.99) than negative control group (0.00 μm ± 0.00) and treated SVF group (654.67 ± 143.78) (Table 2 and Chart 2). Tukey test revealed p values was 0,001, less than 0.05, which means there was a significant difference of mean bone bridge diameter between control positive groups and treated SVF group. Pearson correlation between those two parameters (diameter of bone bridge and mean grey value) demonstrate strong correlation. We can conclude that increasing of mean grey value is proportional to increase of bone bridge diameter from histological analyses. Qualitative histological analysis (Fig. 4) of the SVF group within the defect show regeneration occurred, demonstrated by accumulation of immature chondrocyte that characterized by poorly bordered cell, round nuclei (basophilic) and outside of lacuna. Matrix of growth plate with HE stained shows grey pale colour.

**Fig. 3a:**
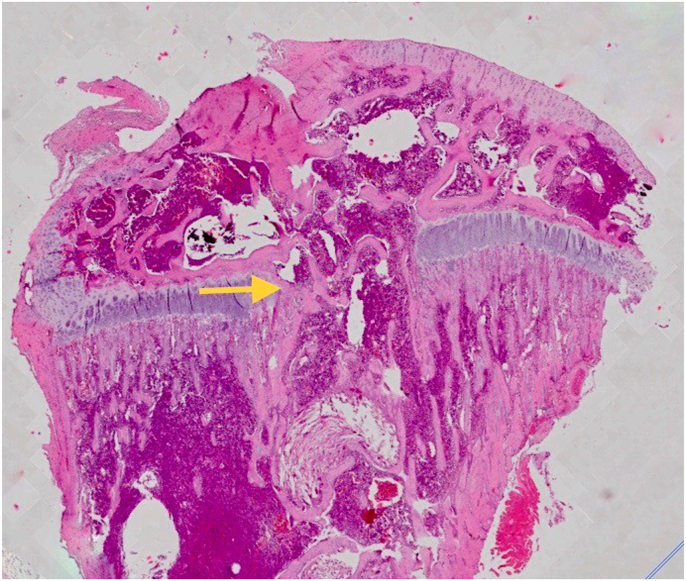
Successful Growth Plate Disruption and Bony Bar Formation. Bony bar formation (yellow arrow) is seen at 28 days post-injury confirmed through hematoxylin-eosin (HE) staining. The bony bar is fully mature by day 28 post-injury. (For interpretation of the references to colour in this figure legend, the reader is referred to the Web version of this article.)

**Fig 3b:**
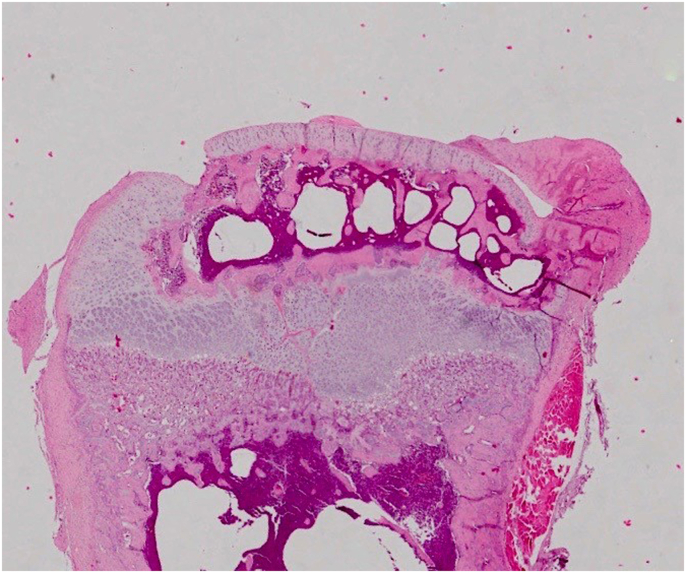
Bony Bridge Formation on Intervention Group. Less Bony Bar formation have been shown in Growth Plate Injury treated with AD-SVF. Diameter of bony bar was measured in 8× magnification of light microscope.

**Table 2:** Descriptive analyses of mean bone bridge diameter between three different groups.

Parameter	Group	N	Mean	Std. Deviation	Minimum	Maximum
DIAMETER (μm)	Kontrol Negatif	6	0.00	0.00	0.00	0.00
	Kontrol Positif	6	1256.83	429.99	778.00	1883.00
	SVF	6	654.67	143.78	444.00	868.00

**Fig. 4:**
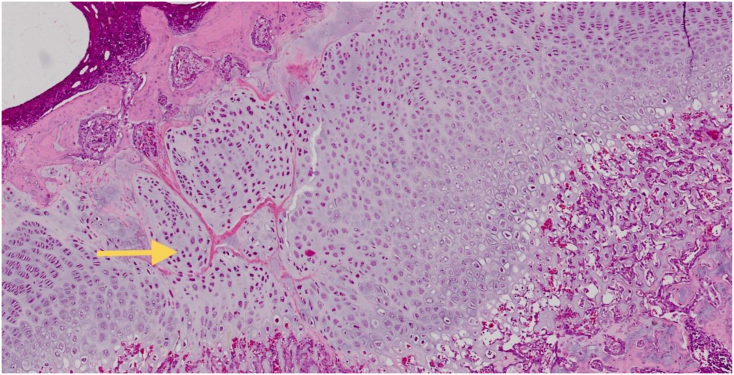
Qualitative histological analysis. The AD-SVF group within the defect show regeneration occurred, demonstrated by accumulation of immature chondrocyte that characterized by poorly bordered cell, round nuclei (basophilic) and outside of lacuna (yellow arrow). Matrix of growth plate with HE stained shows grey pale colour. (For interpretation of the references to colour in this figure legend, the reader is referred to the Web version of this article.)

Chart 2. Descriptive analyzes revealed that mean of bone bridge diameter from positive control group was higher than negative control group and treated SVF group.

SOX 9 analysis reveals that in the positive control group (without SVF) the value was very low when compared to treatment group. Whereas in the treatment group almost similar to the normal value (negative control) data produced were then tested for normality with the results of normal distribution but in the homogeneity test no homogeneous results were obtained so that the subsequent calculations used non-parametric statistical analysis namely Kruskal Wallis and Mann Whitney. Kruskal wallis test showed significant results between groups with p < 0.05. In the Mann Whitney test it was found that the positive control group gave significantly different results from treatment groups. Meanwhile, when compared with normal (negative control) treatment groups did not differ significantly. From immunofloresence analyses was found that in the treatment group the number of osteoblast cells was very small when compared to the positive control where the lowest value was found in the SVF + amnion group with values close to the normal value (Fig. 5b, Fig. 5aa and b). Kruskal wallis calculation showed significant results with p value < 0.05, while the test with Mann Whitney obtained a positive control group significantly different from treatment group. In the control positive group the number of chondrocyte cells was very small (27.67 ± 20.71)when compared to the treatment group (245.83 ± 31.39). The kruskal wallis calculation showed significant results with a p value < 0.05, while the Tukey test found a positive control group significantly different from treatment groups.

**Fig. 5a:**
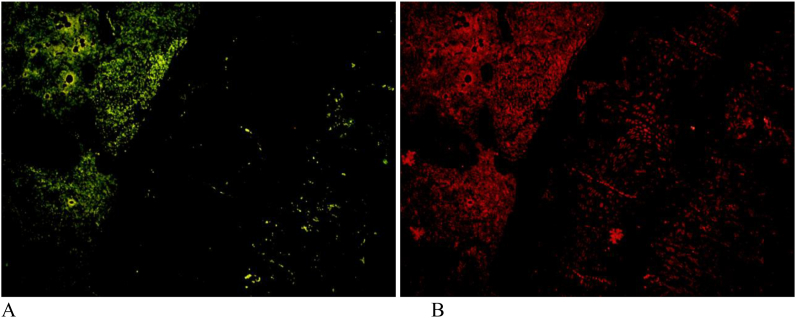
Immunoflorescene (IF) of rat's growth plate area in SVF group. A) IF for osteoblast cell; B) IF for chondrocyte cell.

**Fig. 5b:**
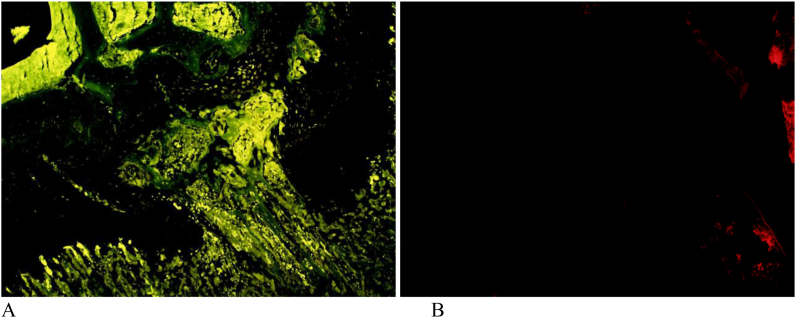
Immunoflorescene (IF) of rat's growth plate area in positive control group. A) IF for osteoblast cell; B) IF for chondrocyte cell.

## 4 Discussion

The aim of this study is to evaluate the potential of Adipose-derived Stromal vascular fraction as a tissue engineering-based therapies for preventing bony bridge formation on growth plate injury model in rat. Previous studies by *Erickson* in 2012 using growth plate injury model in rat have reported bone bridge formation was completed after 28 days following the surgical procedure. In days 28 following surgical procedure, samples were sacrificed and evaluate the amount of bony bridge formation within the defect.

This study evaluates quantitatively the amount of bone bridge diameter by calculating the widest diameter of a growth plate injury defect with histopathological observations stained with hemato-xylin eosin and observed on a light microscope with 8 times and 40 times magnifications. Radiological analyses check the density of the growth plate area using x-ray quantification through ImageJ software obtained from measurements of mean grey values. For qualitative assessments performed on histopathological preparations, the regeneration formed in the injury defect was observed the type of cells that fill the defect whether they have characteristics such as chondrocyte cells or are other cells such as osteoblasts or fibrocytes. ELISA of SOX 9 and immunofloresence analyses also being observed.

Recently, we demonstrated that Intervention group (AD-SVF) has significant less bone bridge formation compare to positive control group. Result shows significant less bone bridge formation in intervention group both histological, radiological analyses, ELISA and immunofloresence. From histopathology analyses in 40× magnification demonstrated immature chondrocyte was found within the defect. Immature chondrocyte is characterized by poorly bordered cell, round nuclei (basophilic) and outside of lacuna. Matrix of growth plate with HE stained shows grey pale colour. So, we concluded that regeneration process was occurred after AD-SVF was injected inside the defect. Fundamentally, the tissue engineering paradigm consists of scaffolds, signals, and cells. These 3 elements can be combined or used independently to attempt to generate tissues in a limitless number of arrangements [11], [12].

The advantage of SVF over ADSCs is believed to be in two fundamental areas. Firstly, although similar in properties such as immunomodulation, anti-inflammatory, angiogenesis, and so forth, the distinctive, heterogeneous cellular composition of SVF may be responsible for the better therapeutic outcome observed in comparative animal studies. Secondly, unlike ADSCs, SVF is much more easily acquired, without the need for any cell separation or culturing conditions. Thus, the therapeutic cellular product is instantaneously obtained and has minimal contact with reagents making it comparatively safer and subject to the fulfilment of lesser regulatory criteria.

In order to create living tissues, as well as integrate living engineered tissue with native host tissues, cells must be present. The presence of AD-MSC (Adipose derived-Mesenchymal Stem Cell) in SVF will increase the number of progenitor cells in the area of growth plate injury. In addition, some of these cells were found to be MSC like as they expressed the stem cell marker alpha-smooth muscle actin. Furthermore, at the early time point day 4 post-injury, some of these cells already expressed chondrogenic marker collagen-2 and/or osteogenic marker Runx 2, suggesting that this influx of mesenchymal cells may contain a myriad of cells including MSC-like cells, osteoprogenitor cells, pre-osteoblasts, and/or pre-chondroblasts (either pre-existing or newly derived from the infiltrated MSCs). Further indicative of potential multipotency for some of these cells, previous studies have shown that differentiation of these cells and growth plate repair outcomes could be influenced by some growth factors or other signals occurring during this phase [4]. Additional MSC and inductive signal molecules for chondrogenesis are important during this phase to reduce bone bridge formation. MSCs are heavily influenced by and require signals (particularly growth factors) for their migration, proliferation and differentiation. In regard to growth plate cartilage repair, ideally, a combination of growth factors would firstly assist with optimal expansion of MSCs and then with the induction of chondrogenic differentiation. In a study conducted by Polly in 2019 regarding the use of SVF (Stromal Vascular Fraction) versus ASC (Adipose stem cells) for tendon healing, it was found that in SVF there was a higher gene expression of several Growth factors including IGF-I, TGF-β1 and FGF-2. Growth factors are growth factors that have a major role in chondrogenesis [7], [8].

TGFβ is a potential induction factor for chondrogenic differentiation from MSC and other mature tissues derived from MSC. Insulin growth factor I (IGF-1) is one of the growth factors that also has a role in repairing growth plates. Its role in chondrocytes/cartilage includes stimulation of extracellular matrix synthesis and a decrease in matrix catabolism except in OA cartilages. IGF-I induces a large number of anabolic effects and reduces catabolic responses. The chondrogenic differentiation of MSCs is induced by IGF-I but is increased when IGF-I and TGF-B1 are used in combination. Fibroblast growth factors (FGF) involved in the process of repairing growth plates is twofold, namely FGF-2 and FGF-18. In MSC, FGF-2 has the role of increasing proteoglycan synthesis and cell proliferation while FGF-18 has no effect.

Stromal Vascular Fraction (SVF) also contains many T regulatory cells (T reg) which are cells in the immunological system. At the growth plate that is injured will release a biomolecule called damage associated molecular pattern (DAMP) which will give effect to the T reg to regulate so that the monocytes turn into macrophages type 2. The more T reg is in the area of the growth plate injury, it is expected there are more and more type 2 macrophages in the area of injury. Type 2 macrophages play a very important role because these type 2 macrophages will initiate a repair process in growth plate injury and have an anti-inflammatory effect that will prevent the occurrence of chronic inflammation which is very detrimental in the ideal healing process. Activation of the pathogenesis pathway through the SoX9 transcription factor will change AD-MSCs to chondrocytes marked by the formation of Col-2a. if this happens it will prevent the occurrence of bone bridge in growth plate injury [6].

From the result we can conclude that AD-SVF prevent the bony bridge formation in injured growth plate in Rat. This study represents the first work that has utilized this rat animal model to investigate whether AD-SVF combine with freeze-dried amniotic membrane can be used to initiate regeneration at the injured growth plate. Questions to be addressed in the future may include analysis of the repair growth plate at different phase of healing process post-operatively to determine the exact mechanism of regeneration process. Second, the potential of MSCs ‘pre-differentiated/committed’ to chondrogenic lineage in vitro prior to transplantation - to improve the possibility of chondrogenic tissue repair. And the last, tracking of the introduced MSCs to determine their role in the repair response - whether MSCs directly contribute to the repair tissue or facilitate the recruitment of endogenous cells. Further study is required to evaluate whether MSC may be a viable therapeutic option for the biological regeneration of the growth plate following injury.

## Annals of medicine and surgery

The following information is required for submission. Please note that failure to respond to these questions/statements will mean your submission will be returned. If you have nothing to declare in any of these categories then this should be stated.

## Registration of research studies

1.Name of the registry:
2.Unique Identifying number or registration ID:
3. Hyperlink to your specific registration (must be publicly accessible and will be checked).

## Appendix A Supplementary data

figs1 figs1
