# Supplementary figures and images for: Adipose-derived stromal vascular fraction prevent bone bridge formation on growth plate injury in rat (in vivo studies) an experimental research
Source: Ann Med Surg (Lond). 2020 Sep 28;60:211–7. doi: 10.1016/j.amsu.2020.09.026 (PMC7645312; doi:10.1016/j.amsu.2020.09.026)

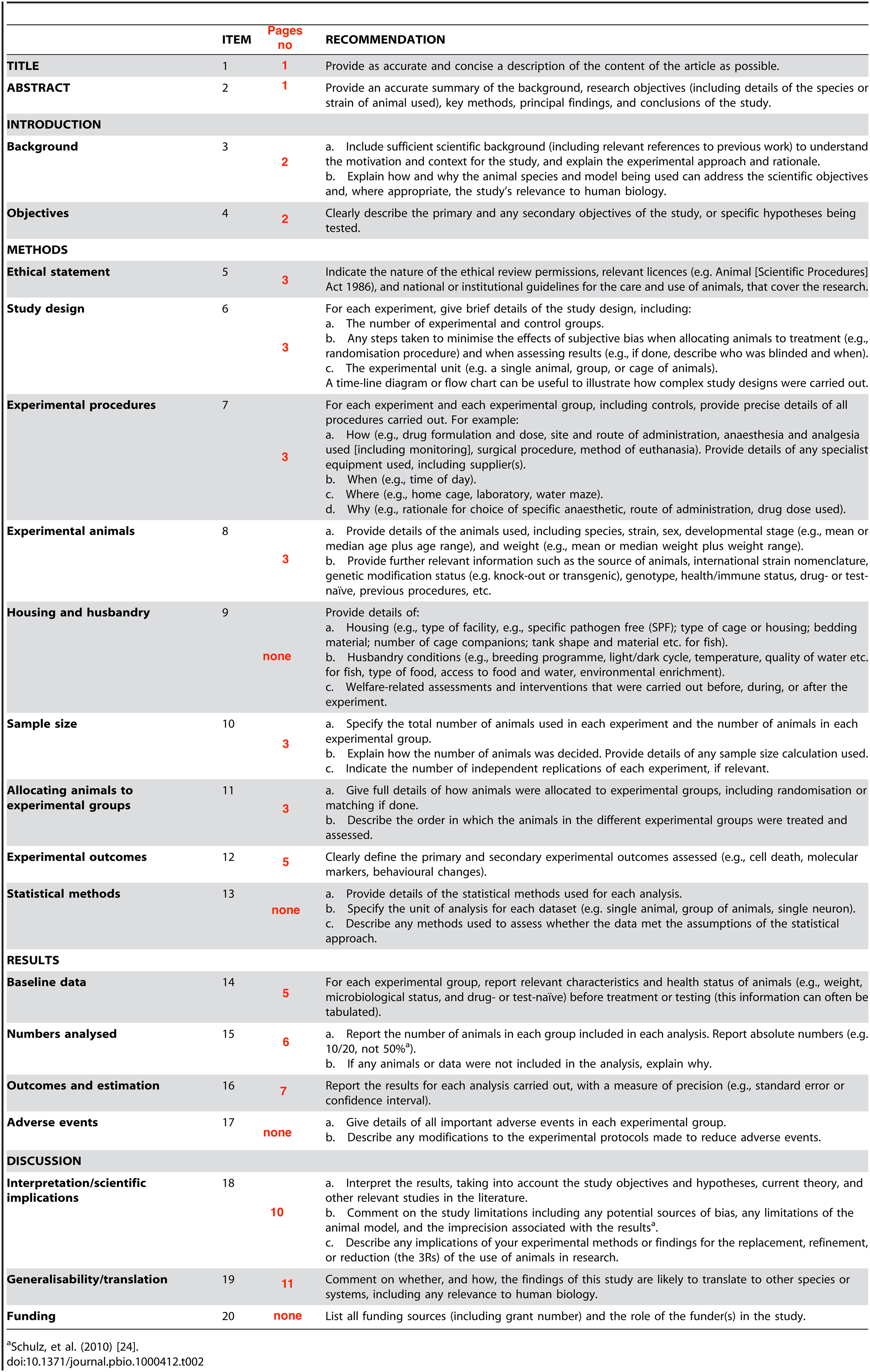

Supplement: figs1 [file mmcfigs1.jpg]
